# Vi-PLUS: Pioneering Plane-Wave Ultrasound to Assess Breast Glandular Tissue in Healthy Women—A Pilot Study

**DOI:** 10.3390/cancers17020237

**Published:** 2025-01-13

**Authors:** Ioana Bene, Delia Doris Donci, Diana Gherman, Manuela Lavinia Lenghel, Carolina Solomon, Ioana-Teofana Dulgheriu, Diana-Raluca Petea-Balea, Cristiana Augusta Ciortea, Larisa Dorina Ciule, Andrada-Larisa Deac, Anca Ileana Ciurea

**Affiliations:** 1 Department of Radiology, Faculty of Medicine, “Iuliu Hațieganu” University of Medicine and Pharmacy, 400012 Cluj-Napoca, Romaniaraluka_jkf@yahoo.com (D.-R.P.-B.);; 2 Department of Radiology, Emergency County Hospital, 400006 Cluj-Napoca, Romaniacristianaciortea@yahoo.com (C.A.C.); 3 Department of Oncology, Emergency County Hospital, 400006 Cluj-Napoca, Romania; 4 Department of Pharmacology, Toxicology and Clinical Pharmacology, Faculty of Medicine, “Iuliu Hațieganu” University of Medicine and Pharmacy, 400012 Cluj-Napoca, Romania

**Keywords:** breast tissue, viscosity, elasticity, ultrasound

## Abstract

Background/Objectives: This study pioneers the application of the ViPLUS module, utilizing plane-wave ultrasound to measure breast tissue viscosity and elasticity. The primary goal was to establish normal reference values for viscosity in healthy women. Secondary objectives included exploring correlations between breast tissue viscosity and breast density categories, hormonal influences, and menstrual cycle phases. Methods: A prospective study was conducted on 245 asymptomatic women. Viscosity and elasticity measurements were obtained using the ViPLUS module, ensuring high reliability with stringent quality control measures. Data were statistically analyzed to evaluate correlations and group differences. Results: The median viscosity value for normal breast parenchyma was 1.7 Pa.s, with no significant variations based on breast density, menopausal status, or menstrual cycle phase. A strong correlation (rho = 0.866, *p* < 0.001) was observed between elasticity and viscosity values. Conclusions: The findings suggest that breast viscosity is consistent across diverse physiological states, indicating its potential as an independent diagnostic marker. This parameter could be pivotal in future breast cancer screening strategies, especially for younger women and those with dense breasts.

## Simple Summary

Recent advances in breast imaging focus on refining diagnostic tools to improve early cancer detection and patient care. This study introduces a novel use of plane-wave ultrasound technology, specifically the Viscosity Plane-wave UltraSound (ViPLUS) module, to evaluate breast glandular tissue. The research aimed to establish normal viscosity values in healthy women, investigate correlations with breast density, menstrual cycle phases, and menopausal status, and explore its potential role in identifying breast cancer risks. The findings could pave the way for personalized breast screening protocols and may lead to significant improvements in diagnostic accuracy, particularly for women with dense breast tissue.

## 1. Introduction

Shear Wave Elastography (SWE) has become a standard method for assessing the parenchymal stiffness of various organs and lesions, providing both qualitative and quantitative measurements. Traditionally, SWE imaging has been based on the assumption that the medium through which the shear wave travels is homogeneous and linear [1]. However, recent advancements have highlighted that biological tissues possess two key mechanical properties—elasticity and viscosity—that significantly affect the transmission of shear waves. These properties alter the relationship between the applied acoustic radiation force and the resulting tissue deformation, a process that is nonlinear and time-dependent [2]. Specifically, elasticity is associated with the speed of the shear wave, which correlates with tissue fibrosis, while viscosity is related to the dispersion of the shear wave, a factor that is notably influenced by inflammatory changes within the tissue. Viscosity represents the intrinsic resistance of tissue to shear deformation, providing insights into the mechanical properties of biological tissues beyond traditional elasticity. Unlike elasticity, which primarily reflects tissue stiffness and correlates with fibrosis, viscosity captures the time-dependent and dissipative properties of tissues, which are heavily influenced by inflammatory and cellular changes. These properties are particularly relevant in breast tissue, where hormonal fluctuations, cellular composition, and structural changes dynamically alter tissue behavior [3].

An increase in necroinflammatory changes within tissues leads to a higher level of shear wave dispersion, which in turn results in elevated tissue viscosity values. This relationship suggests that viscosity could play a significant role in the future of clinical elastographic diagnosis, offering valuable insights for various conditions [4,5]. However, it is important to note that there are only a limited number of studies focusing on viscosity to date, with the majority primarily addressing diffuse liver diseases. In these studies, viscosity values have demonstrated superior diagnostic performance compared to hepatic stiffness, particularly in detecting allograft damage following liver transplantation [6]. Additionally, viscosity levels have been found to correlate with the degree of lobular inflammation in patients diagnosed with non-alcoholic fatty liver disease [7].

Despite its potential, viscosity remains underexplored in the context of breast imaging. Viscosity, as a measurable parameter, could reveal subtle changes in tissue composition and microenvironment not captured by traditional imaging modalities. For instance, elevated viscosity may indicate increased interstitial fluid content, cellular proliferation, or necroinflammatory activity, all of which are indicators of pre-cancerous or cancerous conditions [3].

The primary goal of our study is to establish a reference value for the viscosity of breast parenchyma. Our secondary objectives include investigating the potential correlation between viscosity values and breast density categories, exploring whether there are differences in breast viscosity between premenopausal and postmenopausal women, and determining if breast viscosity varies across different phases of the menstrual cycle.

## 2. Materials and Methods

### 2.1. Population

We conducted a prospective single institutional study between November 2021 and February 2022 that included a total of 245 patients. All patients were asymptomatic and were addressed to our department for screening examinations. For women aged 40 years and older mammography and breast ultrasounds were performed, whereas in women younger than 40 years old breast ultrasounds alone was performed. The results were structured using the standard Breast Imaging Reporting and Data System (BI-RADS).

Patients were included in the study if they had no abnormalities in the clinical exam performed by the radiologist and if they had either negative breast examinations classified BI-RADS 1 or benign breast findings classified BI-RADS 2 such as simple cysts, intramammary lymph nodes, cytosteatonecrosis, fibroadenomas that had previously been followed for more than 2 years, lipomas and calcifications with benign morphology. The exclusion criteria were the following: patients receiving hormone replacement therapy or hormonal contraception, patients with a history of breast cancer, and patients who experienced the onset of menopause less than one year before examination.

The reproductive status (menstruating or menopausal) and, for women of reproductive age, the phase of the menstrual cycle at the time of examination were also recorded. Furthermore, patients of reproductive age who were first seen on day 7 or day 21 of their menstrual cycle were called for a second examination following the same protocol two weeks later. Therefore, we obtained two different breast assessments 7 days before and 7 days after the onset of menstruation.

### 2.2. Ultrasound Acquisition Technique

The ultrasound was performed using a SuperSonic MACH^®^ 30 machine (Aixplorer, SuperSonic Imagine, Aix-en-Provence, France). All measurements were acquired by a radiologist with 7 years of experience in breast ultrasonography, who was trained for 5 days in performing viscosity measurements.

The patients were positioned supinely, with the arms abducted and the hands placed beneath the head. Firstly, a standard ultrasound of the two breasts and axilla was performed using a linear L18-5X probe to assess the glandular tissue distribution within the breast and to confirm the absence of any pathologic finding. Subsequently, measurements of stiffness and viscosity were obtained in the upper-outer quadrant of the right breast. These two measurements were acquired simultaneously using the ViPLUS module, which was available only on a curvilinear C6-1X probe. The curvilinear probe was placed on the skin surface and an optimal gray-scale image was obtained. The curvilinear transducer was utilized because the ViPLUS module was exclusively available on this type of transducer. However, the primary goal of this preliminary study was to gather quantitative data on viscosity rather than to evaluate parenchymal structural changes, which require higher-frequency transducers. Additionally, the images obtained achieved a stability index (SI) exceeding 90%, enabling optimal measurements. SI, a relatively new parameter developed by Supersonic Image, reflects the spatial and temporal stability of tissue rigidity within a circular Q box. Further studies using linear transducers are needed to validate the normal viscosity values obtained in this study.

**Figure 1:**
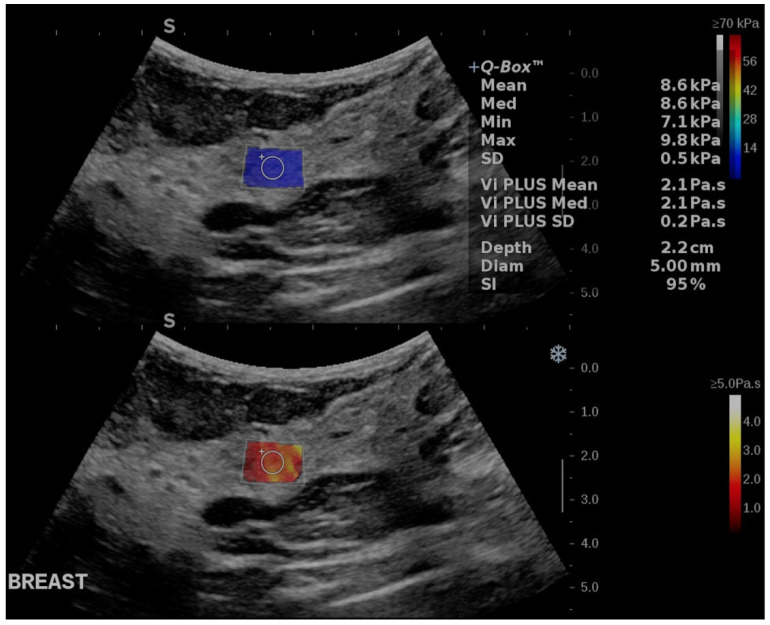
Quantitative measurements made on breast glandular tissue, obtaining viscosity (blue box) and elasticity (red box).

Thereafter, without applying any external compression, the ViPLUS module was activated. The ViPLUS Q box was placed within the breast tissue and a region of interest with a diameter of 5 mm was set at a depth of approximately 2 to 3 cm underneath the skin. After obtaining a homogenous signal in the Q box, a color-coded map was available for the qualitative assessment of both elasticity and viscosity. Quantitatively, the elasticity measurements were expressed in kilopascals (kPa) and viscosity values were expressed in Pascal.second (Pa.s). Regarding viscosity, measurements were considered valid if the internal parameter for quality control, the stability index (SI), exceeded 90% and if the standard deviation was lower than 10% of the mean viscosity value registered (Figure 1). Three measurements were acquired for each patient and the resulting average value was retained for statistical analysis.

### 2.3. Statistical Analysis

Statistical analysis was performed using MedCalc Version 20 (MedCalc Software Corp., Brunswick, ME, USA). The Shapiro–Wilk test was used to evaluate data distribution.

The quantitative variables were expressed as median values and interquartile range (25–75% percentiles) for non-normally distributed data and as mean values and standard deviation for normally distributed data. The Spearman rank correlation (rho) was employed for assessing the relationship between elasticity and viscosity values. Comparison between two independent groups with non-normal data distribution was assessed by applying the Mann–Whitney test, while the Kruskal–Wallis test was employed for comparisons among multiple independent groups with non-normal data distribution. The Wilcoxon test was used for paired groups with non-normal data distribution. Results were deemed statistically significant when a *p*-value < 0.05 was obtained.

## 3. Results

**Figure 2:**
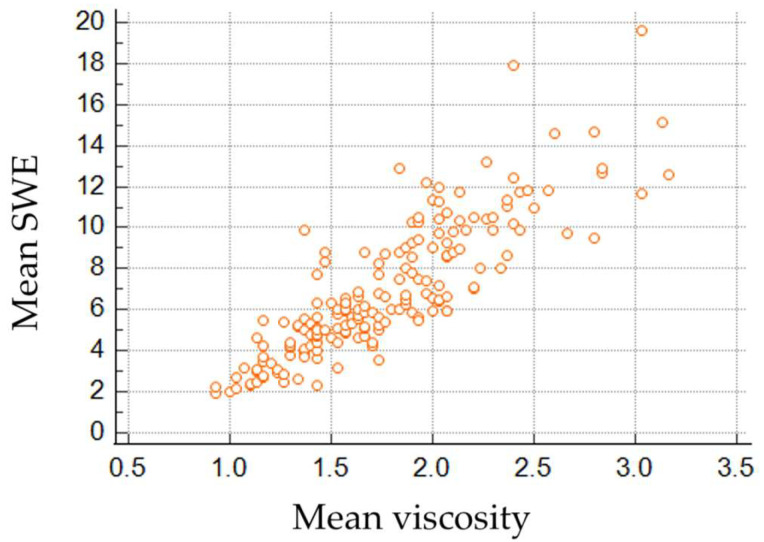
Spearman rank correlation indicates a strong correlation between SWE and viscosity values.

A total of 245 patients were included in the study, of whom 179 patients underwent both mammography and ultrasound examinations, while 66 patients underwent only ultrasound examination. The mean age was 48 years. The SWE and viscosity values of normal breast parenchyma had a non-normal distribution. The overall median and 25–75% percentiles for elasticity were 6.03 (4.65, 9.02) kPa, and for viscosity were 1.7 (1.43, 2.03) Pa.s. A strong and direct proportional correlation between SWE and viscosity values was recorded (rho of 0.866, *p* < 0.001) (Figure 2).

**Figure 3:**
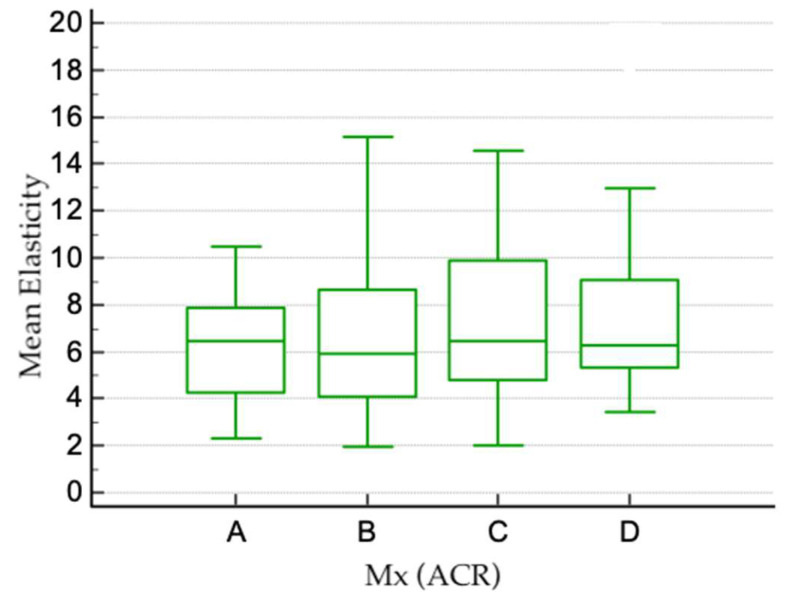
Boxplot showing the mean values of tissue elasticity for each type of breast density.

**Figure 4:**
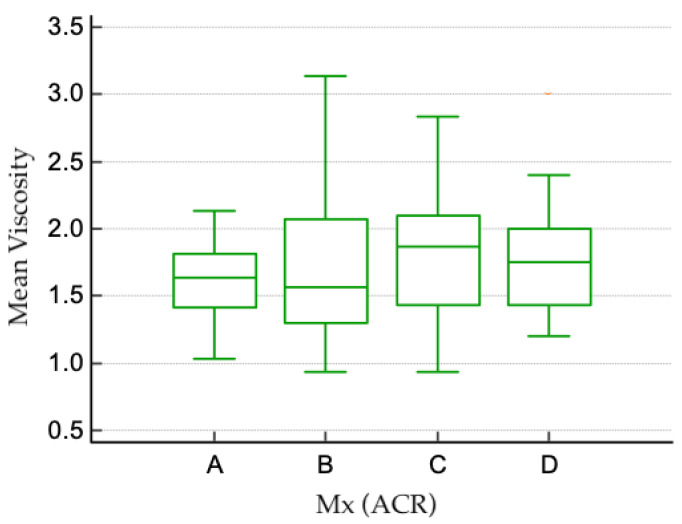
Boxplot showing the mean values of viscosity for each type of breast density.

For the 179 patients who underwent both mammographic and ultrasound examinations, a possible correlation between mammographic American College of Radiology (ACR) breast density categories and the values of SWE and viscosity was analyzed. Measurements obtained for each type of breast density are shown in Table 1, Figure 3 and Figure 4. No statistically significant difference was found between SWE or viscosity values in different ACR types of breasts (Kruskal–Wallis test for SWE *p* = 0.305 and viscosity *p* = 0.099).

SWE and viscosity breast values were compared between women of reproductive age and postmenopausal women using the Mann–Whitney test. No significant difference was found between these two groups of patients (*p* = 0.428 for SWE and *p* = 0.992 for viscosity) (Table 2).

Finally, 33 patients of reproductive age underwent an ultrasound examination on day 21 of their menstrual cycle and a second one on day 7 after menstruation onset. There were no significant differences in elasticity and viscosity measurements between breasts in different menstrual cycle phases (Wilcoxon test for SWE with *p* = 0.386 and for viscosity with *p* = 0.68) (Table 3).

## 4. Discussion

All of the imaging techniques currently available for evaluating the mammary gland have specific advantages and limitations, none of them having 100% sensitivity or specificity. Breast diagnosis requires, in almost all cases, the combination of several imaging techniques. There are also currently imaging characteristics that can identify patients at increased risk of developing breast cancer, namely glandular density in mammography [8,9,10,11] or the intensity of uptake of the contrast agent by the glandular parenchyma in magnetic resonance examination (MRI) [12,13,14]. However, these characteristics cannot be widely used for risk stratification and risk classification of the female population considering that mammographic screening begins at the age of 45–50 years (depending on the country) and MRI examination is recommended for screening purposes only in high-risk patients and for diagnostic purposes only as an evaluation method in a more advanced stage of diagnosis of breast cancer patients.

For this reason, finding additional assessment modalities that identify intermediate-risk patients or allow early diagnosis without the use of contrast agents would find practical utility in both screening and diagnosis.

ShearWave Elastography (SWE) is routinely used to quantify parenchymal stiffness of multiple organs and lesions, both qualitatively and quantitatively. Until recently, SWE imaging techniques assumed that shear wave transmission occurs in a homogeneous and linear medium [1]. However, biological tissues are characterized by two mechanical properties, elasticity and viscosity, both of which influence the relationship between the delivered acoustic radiation force and tissue deformation, which are nonlinear and time-dependent [2]. Elasticity reflects shear wave velocity, which is influenced by tissue fibrosis, while viscosity reflects shear wave dispersion, which is influenced by inflammatory changes [3]. To date, only a few studies have been performed that evaluated viscosity as a new imaging parameter, mainly in chronic liver diseases [4,15].

There are a few studies evaluating viscosity in soft tissues. Muntean et al. [16] evaluated and established the normal values of the parotid (PG) and submandibular (SMG) gland viscosity. They concluded that the normal mean value of parotid gland viscosity is 2.13 ± 0.23 Pa.s, significantly lower compared to the mean value of submandibular gland viscosity of 2.44 ± 0.35 Pa.s (*p* < 0.05). This difference could be explained in part by the glandular structure, the PG being a predominantly serous gland, which secretes watery saliva, while the SMG is a mixed muco-serous gland, producing more viscous saliva [17]. Viscosity values did not show statistically significant differences according to sex, and regarding the age of the subjects, the authors found a minimal difference in viscosity values for the submandibular glands.

In another study, functional variations in the parotid (PG) and submandibular glands (SMG) were evaluated by determining the values of elasticity and viscosity before and after the administration of a sialagogue agent [18]. The study concluded that under basal conditions, parotid glands exhibit significantly higher elasticity and significantly lower viscosity compared to submandibular glands. After administration of the sialagogue, the elasticity and viscosity values increased significantly for the parotid glands. For the submandibular glands, although the values were higher post-stimulation, no statistically significant differences were found between the basal and post-stimulation values. There were also no statistically significant differences between the pre-and poststimulation values of elasticity and viscosity related to the subjects’ gender or body mass index.

Dulgheriu et al. [19] aimed to establish normal reference values of peripheral muscle viscosity, at rest and after exercise (postcontraction), as well as interobserver variability. They found that the elasticity and viscosity were significantly higher for the deltoid muscle compared to the soleus muscle, both at rest and post contraction. As well, the values measured at rest and post contraction showed statistically significant differences both for the soleus muscle and for the deltoid muscle.

Although we initially anticipated that certain differences would emerge due to various biological factors affecting breast tissue, including the distinct proliferative changes associated with breast type, patient age, and menstrual cycle phases, our findings did not reveal any statistically significant differences across viscosity and ACR breast type, viscosity before and after menstruation (luteal and proliferative phases) or women of reproductive age and women in menopause. We hypothesized that breast tissue viscosity might vary based on breast type, considering that different breast types can exhibit unique structural and cellular characteristics, potentially affecting fluid properties. However, our analysis showed no significant differences in viscosity measurements among the various breast types examined, although previous studies have demonstrated positive correlations between ultrasound elasticity and mammographic density [20]. Furthermore, given the physiological changes during the menstrual cycle, with hormone levels fluctuating between the luteal and proliferative phases, we expected viscosity to be influenced by these hormonal shifts. Specifically, the luteal phase is typically associated with increased glandular activity, while the proliferative phase is characterized by cellular regeneration and growth [21]. Despite these cyclical changes, our study found no observable differences in viscosity between these phases.

It is well-documented that hormonal changes significantly affect breast tissue as women age, particularly in the transition from reproductive years to menopause when, due to the lack of estrogenic activity and the involution of the mammary gland, the reduction in/absence of proliferative changes might impact viscosity in terms of obtaining lower viscosity values in the menopause period [22]. Nevertheless, our results indicated no statistically significant differences in viscosity between women of reproductive age and those in menopause.

These findings suggest that contrary to our initial expectations, breast tissue viscosity remains consistent across various physiological changes related to age, menstrual cycle, and breast type. We found that the normal viscosity of the mammary gland is 1.76 ± 0.46 Pa.s. and found a positive correlation between SWE and ViPLUS, an aspect commonly observed in studies conducted on various anatomical regions [19,23].

Preliminary data from the study described above suggest that viscosity does not depend on breast type or hormonal status and has a standard normal value in healthy patients. As a result, viscosity could represent an independent predictive factor for the development of breast cancer, but first it requires further studies in order to find a correlation between viscosity values and the presence of breast cancer. A follow-up study would be valuable to test this hypothesis and assess whether there are significant differences in the elasticity and viscosity values of the mammary gland between healthy individuals and breast cancer patients.

If our hypothesis is verified, it will have an important impact on methods of breast screening. At the moment it is proven that patients with dense breasts have a higher risk of developing breast cancer, which is why, in the USA, these patients benefit from ultrasound screening in addition to mammography; ultrasound screening practically doubles the number of cancers detected [24,25,26,27]. However, this screening is only aimed at patients over 50 years of age and younger patients, even if they have a family history of breast cancer, are not included in the screening programs unless they present genetic mutations [28,29]. These patients, at the time of diagnosis of a first-degree relative with breast cancer, could have an ultrasound scan that includes the measurement of the elasticity and viscosity values of the mammary gland and, if these values are changed compared to the reference values, a personalized screening protocol could be established for them.

Based on our results, we propose that the lack of significant differences observed between groups suggests that viscosity remains relatively stable in healthy breast tissue, regardless of variations in factors such as breast density, menopausal status, or hormonal phases. This stability indicates that viscosity may not exhibit substantial variability within the normal physiological range. Furthermore, establishing viscosity profiles for patients with breast cancer could be valuable, providing a potential biomarker for early detection and improved risk stratification in breast cancer care.

This study has several limitations. First, it was a single-center study and the measurements were conducted using a curvilinear transducer, as the Vi.PLUS mode is currently only available on this type of transducer. However, reliable measurements were achieved with a Stability Index exceeding 90%, which aligns with the manufacturer’s quality standard. The focus of this study was to gather quantitative data on viscosity and elasticity values, rather than assess structural changes in the breast glandular parenchyma, which would require the use of high-frequency transducers.

Future perspectives on developing the ViPLUS module for breast cancer patients could include:

1. Integration into breast cancer risk assessment.

The ViPLUS module could be further developed to serve as an integral part of breast cancer risk assessment. By establishing distinct viscosity values for benign, pre-cancerous, and malignant lesions, this technique may enhance the accuracy of early detection and risk stratification, especially for women with dense breast tissue who are at a higher risk of breast cancer.

2. Correlation with tumor microenvironment changes.

Future research can explore the relationship between breast tissue viscosity and the tumor microenvironment, including factors like fibrosis, angiogenesis, and inflammation. Understanding these correlations could help in identifying specific tissue characteristics associated with malignancy, allowing for improved diagnostic precision.

3. Enhancing screening protocols for younger patients.

Currently, younger women are often excluded from routine breast cancer screening unless they have significant risk factors. The ViPLUS module could be tailored for use in this demographic by providing a non-invasive, radiation-free, and cost-effective alternative for identifying abnormal viscosity patterns that might indicate early cancer development.

4. Monitoring of treatment response.

Viscosity measurements could be employed to monitor changes in breast tissue over time in patients undergoing cancer treatment. By assessing how viscosity evolves in response to therapies like chemotherapy, radiation, or hormonal treatments, the method could serve as a non-invasive tool to evaluate treatment efficacy and disease progression.

5. Development of high-resolution transducers.

Enhancements in the ViPLUS technology could include adapting high-frequency transducers to allow for precise localization of abnormalities within the breast. This advancement could be particularly useful for detecting small, early-stage tumors or tracking tumor margins during treatment, potentially improving outcomes for breast cancer patients.

Each of these perspectives underscores the potential of ViPLUS as an innovative tool in the diagnosis, management, and treatment of breast cancer.

## 5. Conclusions

Preliminary findings from the study indicate that breast viscosity is not influenced by breast type or hormonal status and maintains a consistent normal value in healthy individuals. Therefore, viscosity may serve as an independent predictive factor for the development of breast cancer.

## Figures and Tables

**Table 1:** SWE and viscosity values for each type of breast density according to the ACR mammographic classification of breasts.

Type of Breast Density	No. Patients	SWE (kPa)	Viscosity (Pa.s)
A	16	6.46 (4.25, 7.88)	1.63 (1.41, 1.81)
B	59	5.9 (4.08, 8.65)	1.56 (1.3, 2.06)
C	78	6.45 (4.8, 9.86)	1.86 (1.42, 2.1)
D	26	6.26 (5.3, 9.03)	1.75 (1.43, 2)

Values are expressed as median and 25–75% percentiles.

**Table 2:** SWE and viscosity values based on the reproductive status of patients.

Reproductive Status	No. Patients	SWE (kPa)	Viscosity (Pa.s)
Reproductive age	103	5.7 (4.65, 8.82)	1.66 (1.43, 2.05)
Menopause	142	6.45 (4.6, 9.3)	1.75 (1.43, 2.06)

Values are expressed as median and 25–75% percentiles.

**Table 3:** SWE and viscosity values based on the phase of the menstrual cycle in women of reproductive age.

Phase of Menstrual Cycle	SWE (kPa)	Viscosity (Pa.s)
Premenstrual	6.1 (4.92, 8.95)	1.7 (1.43, 2.16)
Postmenstrual	6.6 (4.5, 10.7)	1.73 (1.53, 2.33)

## Data Availability

The original contributions presented in the study are included in the article; further inquiries can be directed to the corresponding author.
